# A prospective feasibility study evaluating the implementation of model-informed precision dosing in critically ill children

**DOI:** 10.3389/fphar.2026.1733291

**Published:** 2026-02-03

**Authors:** Izgi Bayraktar, Merve Kaşıkcı, Zuhal Benek, Karel Allegaert, Berna Egehan Oruncu, Selman Kesici, Benan Bayrakci, Nadir Yalcin

**Affiliations:** 1 Department of Clinical Pharmacy, Faculty of Pharmacy, Hacettepe University, Ankara, Türkiye; 2 Department of Biostatistics, Faculty of Medicine, Hacettepe University, Ankara, Türkiye; 3 Department of Pharmaceutical and Pharmacological Sciences, KU Leuven, Leuven, Belgium; 4 Department of Development and Regeneration, KU Leuven, Leuven, Belgium; 5 Child and Youth Institute, KU Leuven, Leuven, Belgium; 6 Department of Clinical Pharmacy, Erasmus MC, Rotterdam, Netherlands; 7 Department of Pediatric Intensive Care Medicine, Life Support Center, Hacettepe University, Ankara, Türkiye

**Keywords:** aminoglycoside, children, critically ill, glycopeptide, model informed, precision dosing

## Abstract

Achieving optimal antibiotic exposure in critically ill pediatric patients is difficult due to (their) dynamic physiology and variability. Conventional weight-based regimens often fail to reach pharmacokinetic/pharmacodynamic (PK/PD) targets for narrow therapeutic index agents such as vancomycin and amikacin. Model-Informed Precision Dosing (MIPD), which integrates Bayesian forecasting with population pharmacokinetics (popPK), offers a potentially valuable yet underexplored approach in pediatric intensive care to better attain and sustain target exposure. This study was designed as a prospective, pragmatic feasibility study with a comparator arm, employing observational analyses to evaluate the implementation and methodological performance of MIPD in a tertiary pediatric intensive care unit. Pediatric patients receiving vancomycin or amikacin were managed either with MIPD-guided dosing using a clinical decision support platform or with standard-of-care (SoC) dosing. Primary outcomes included prediction accuracy (*a priori* vs. *a posteriori*) and model fit, defined by the agreement between observed and model-predicted concentrations and categorized as poor, intermediate, or good. Secondary outcomes assessed dose optimization, inflammatory response, renal safety, treatment duration, and mortality. Forty-one patients (median age 38.6 months) were enrolled; Patients were initially allocated to either MIPD or SoC; however, the final analytic groups were defined as-treated, with 12 receiving active MIPD-guided dose adjustments and 29 managed under SoC dosing. Some baseline differences were observed between groups, particularly in inflammatory markers. Clinical outcomes were similar between groups, with numerically greater but nonsignificant reductions in CRP and procalcitonin in the MIPD group. However, these findings were confounded by baseline imbalances and should be interpreted cautiously. Model fit remained unchanged in the SoC group but showed modest improvement for vancomycin under MIPD. Larger, multicenter trials are warranted to confirm clinical benefit and optimize implementation in pediatric intensive care.

**Clinical Trial Registration:**

ClinicalTrials.gov, identifier NCT07315438.

## Introduction

Antibiotics are among the most frequently prescribed medications in hospitalized children, yet their dosing remains particularly challenging due to the complex and variable pharmacology in pediatric populations (Poole et al., 2019; Gijsen et al., 2021). Optimal antibiotic therapy in children is a critical area of clinical care that requires careful consideration of both pharmacokinetic (PK) and pharmacodynamic (PD) properties of the drug, as well as patient-specific factors (Abdulla et al., 2021). In pediatric populations—especially in critically ill children—age-related physiological changes, growth, development, as well as their underlying conditions such as burns, cancer, extracorporeal support or renal failure significantly influence antibiotic PK and PD (Tanaka et al., 2025).

These factors contribute to the high interindividual variability in drug exposure, often rendering conventional weight-based dosing regimens insufficient (Tanaka et al., 2025; Ewoldt et al., 2022). Suboptimal antibiotic exposure, including both underexposure and overexposure, may lead to treatment failure, antimicrobial resistance, or concentration-dependent toxicity (e.g., nephrotoxicity or ototoxicity) respectively. For example, aminoglycosides (such as amikacin) (Smits et al., 2017) and vancomycin (De Cock et al., 2024) possess narrow therapeutic window, necessitating precise dose adjustments to ensure both efficacy and safety (De Cock et al., 2024). Moreover, although not fully elucidated, underexposure of antibiotics may contribute to the emergence of resistant pathogens.

Traditional pediatric antibiotic dosing is based on anthropometric measurements or nomograms and adjusted for renal function but often fails to achieve PK/PD targets (Abdulla et al., 2021). While therapeutic drug monitoring (TDM) helps optimize exposure and reduce toxicity, it is typically performed at steady state, delaying early intervention. In critically ill children, timely and precise dosing is essential (Roberts et al., 2012).

To address this limitation, early TDM sampling combined with population pharmacokinetic (popPK) modeling has emerged as a valuable strategy to estimate drug exposure even before steady-state is achieved. This approach interprets serum concentrations in conjunction with patient-specific characteristics such as age, body weight, renal function, and dosing history (Pea et al., 2002). While popPK modeling and TDM enhance individualized therapy, they primarily inform dose adaptation based on observed concentrations.

To further overcome these limitations and improve early-phase interventions, Model-Informed Precision Dosing (MIPD) strategies have gained increasing attention (Tanaka et al., 2025). MIPD integrates popPK models with Bayesian forecasting to estimate individual PK parameters using measured serum concentrations and patient demographics (e.g., age, weight, height, renal function). This allows for early and individualized dose adjustment, addressing the shortcomings of traditional TDM approaches that rely on steady-state sampling (Smits et al., 2015). Through MIPD, optimized drug exposure targets—such as the area under the concentration-time curve to minimum inhibitory concentration ratio (AUC/MIC), peak concentration to MIC ratio (Cmax/MIC), or the percentage of time the free serum concentration remains above MIC (%fT>MIC)—can be more reliably achieved (Tanaka et al., 2025).

MIPD has shown promising results in pediatric populations, particularly for antibiotics with narrow therapeutic window like vancomycin and amikacin (Smits et al., 2017). However, the widespread implementation of MIPD in clinical settings and its definitive impact on clinical outcomes remain areas of ongoing research (Van Wynsberge et al., 2024).

This study aimed to evaluate the feasibility, implementation characteristics, and methodological performance of MIPD in pediatric intensive care unit (PICU) patients treated with vancomycin or amikacin. The primary objective was to assess within-patient predictive performance by comparing *a priori* and *a posteriori* model-predicted and observed serum concentrations, including evaluation of prediction accuracy and model fit. Secondary objectives were explored descriptively and included characterization of dose adjustments, biomarker trajectories, and safety parameters and were considered exploratory and hypothesis-generating to inform future studies.

## Methods

This study was conducted at a tertiary referral care hospital, which is the largest children’s hospital in Türkiye. The study was designed as a prospective, pragmatic feasibility study with a comparator arm, conducted between December 2023 and June 2024. Also, the study protocol was approved by the Local Ethics Committee, and written informed consent was obtained from the parents or legal guardians of all participants.

### Study allocation and analytic group definition

Each eligible pediatric patient was initially allocated in a 1:1 ratio to either the SoC group or the MIPD-guided dosing group. However, in a subset of patients initially allocated to the MIPD arm, the model-recommended dose was identical to the standard-of-care regimen at baseline and remained unchanged after incorporation of the first measured concentration. As no active model-informed dose adjustment occurred at any point during therapy, these patients were pragmatically analyzed under the SoC arm. Consequently, final analytic groups were defined based on actual exposure to model-informed dosing rather than initial group allocation. This approach ensured that only patients receiving an actual model-guided intervention were included in the MIPD group. To minimize performance bias, pediatric residents responsible for entering medication orders were unaware of patient group assignments. Since routine clinical pharmacy services had already been implemented in the unit for the past 2 years prior to the initiation of the MIPD program, the MIPD-driven recommendations were perceived as part of routine clinical pharmacy practice. This approach minimized the risk of a Hawthorne effect during the allocation process.

### Study population and inclusion/exclusion criteria

The study included pediatric patients admitted to the PICU who initiated treatment with vancomycin or amikacin and underwent TDM. Exclusion criteria included failure to obtain written informed consent, death within the first 24 h prior to treatment initiation, unsuitability as judged by the treating physician, or discontinuation of therapy before the first TDM measurement.

**FIGURE 1:**
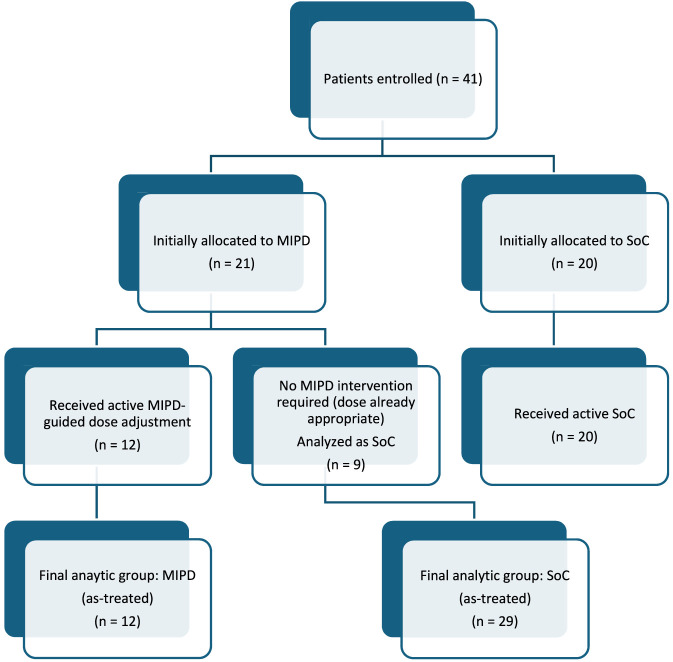
Study flow diagram illustrating patient allocation, receipt of MIPD intervention, and final as-treated analytic groups.

Patient allocation, receipt of model-informed precision dosing intervention, and final as-treated analytic groups are summarized in Figure 1. Although each eligible patient was initially allocated to a treatment arm, they were finally analyzed based on their actual exposure to model-guided interventions (initially allocated vs. finally analyzed as-treated).

### Sample size

The study was primarily exploratory, aiming to evaluate methodological feasibility rather than to achieve statistical power for clinical endpoints. The sample size was determined based on the expected number of eligible PICU patients within the study period and was considered adequate for exploratory analyses of feasibility and model performance.

### Model-informed precision dosing procedure

#### MIPD-guided dosing workflow

MIPD was performed using InsightRX Nova® (*version 1.63.9 or later, San Francisco, California*), a cloud-based clinical decision support platform designed to individualize drug dosing through the integration of popPK models, Bayesian forecasting, and patient-specific clinical data. The platform enables real-time prediction of drug exposure and supports individualized dose optimization in diverse clinical settings. Access to InsightRX Nova® was granted with permission from the developers for research purposes without integration to the electronic health record system.

For each patient, demographic and clinical parameters—including age, weight, height, and serum creatinine—were entered into the platform. InsightRX then applied validated published and validated population pharmacokinetic models with predefined structural assumptions and covariate relationships to estimate pharmacokinetic parameters and predict individualized drug concentrations (Sherwin et al., 2014). Bayesian updating was performed using informative priors derived from these reference models. Dose recommendations were generated to achieve target therapeutic window, including an AUC/MIC ratio of ≥400 for vancomycin, with a suggested trough concentration range of 10–15 mg/L (De Cock et al., 2024), and a Cmax/MIC ratio of ≥8–10 for amikacin, with a target trough (minimum) serum concentration of <5 μg/mL (Illamola et al., 2018). For vancomycin, AUC-based targets were prioritized for MIPD-guided dose optimization, while trough concentrations were used as supportive safety and monitoring parameters to maintain consistency with routine clinical practice.

Model-informed precision dosing was supported by previously published population pharmacokinetic models developed in pediatric populations with substantial representation of critically ill patients. Amikacin dosing was informed by a model developed by Sherwin et al. (2014) in hospitalized children aged 6 months to 17 years treated in a high-acuity burn unit, a population characterized by severe illness and augmented renal clearance. The model used a two-compartment nonlinear mixed-effects structure, with body weight identified as the primary covariate influencing clearance and volume of distribution.

Vancomycin dosing was guided by a population pharmacokinetic model developed by Le et al. (2014) using data from pediatric patients aged 3 months to 21 years, approximately half of whom were managed in intensive care units. This model incorporated age, body weight, and serum creatinine as key covariates and was specifically developed to support AUC-guided dosing strategies in critically ill children. Together, these models were selected to ensure applicability to the PICU setting and to support individualized dosing in patients with high pharmacokinetic variability.

In the present study, model structure, population parameter estimates, interindividual variability terms, residual error models, and covariate relationships were implemented as reported in the original publications and were not re-estimated. These model components were treated as prior information within the Bayesian framework. Bayesian updating was applied exclusively to individual pharmacokinetic parameters using observed serum concentrations obtained during routine therapeutic drug monitoring. Key structural and variability features of the population pharmacokinetic models used for Bayesian forecasting are summarized in Table 1.

**TABLE 1:** Population pharmacokinetic models used for Bayesian forecasting.

Drug	Structural model	Parameters	Covariates	IIV^a^	Residual error	References
Vancomycin	One-compartment	CL, V	Weight, age, serum creatinine	CL, Vd	Not defined	Le et al. (2014)
Amikacin	Two-compartment	CL, V1, V2, Q	Weight	CL	Combined (additive + proportional)	Sherwin et al. (2014)

CL, clearance; V, volume of distribution; V1, central volume of distribution; V2, peripheral volume of distribution; Q, intercompartmental clearance; IIV, interindividual variability. Residual error refers to the statistical error model used to describe unexplained variability in observed concentrations. Combined error indicates the inclusion of both additive and proportional error components.

^a^ IIV, indicates parameters for which interindividual variability was estimated in the original model.

For standardization purposes, a default MIC value of 1 mg/L was assumed for both vancomycin and amikacin. Derived PK/PD indices (AUC/MIC and Cmax/MIC) were calculated for comparative and descriptive purposes only and were not used to define target attainment or clinical efficacy. We hereby stress that these, AUC/MIC ratios in this study should be interpreted as relative exposure metrics rather than absolute indicators of clinical target attainment. For amikacin, although EUCAST defines a clinical susceptibility breakpoint of 4–8 mg/L, a nominal MIC value of 1 mg/L was assumed for simulation purposes to maintain consistency with prior pharmacokinetic modeling studies and to enable standardized comparison across antibiotics (Le et al., 2014; Sherwin et al., 2014). This assumption does not represent a clinical breakpoint. All exposure metrics, including AUC and Cmax, were model-derived estimates generated by the MIPD platform based on population pharmacokinetic models and Bayesian updating, rather than directly measured concentrations.

To address whether incorporating TDM data improves predictive performance, we generated *a priori* predictions (covariate- and dosing-based, without assimilating any measured concentrations) and *a posteriori* prediction (after assimilating the first measured concentration) in InsightRX for each patient. Predictive accuracy was evaluated by comparing observed levels against *a priori* and *a posteriori* prediction at the first and (when available) second sampling time points.

All MIPD simulations were conducted by a clinical pharmacist trained in pharmacometrics and therapeutic drug monitoring, and the use of the specific software program. Dosing recommendations generated by InsightRX were communicated to the attending pediatric intensivists during routine clinical rounds. Physicians retained full authority over the final prescribing decisions, and MIPD-derived suggestions were implemented only after verification of clinical appropriateness, safety limits, and consistency with institutional protocols. Therefore, while the software recommendations informed dose optimization, their adoption was not mandatory but subject to clinician approval.

To ensure patient safety, all software-generated dose recommendations were reviewed before implementation. Specifically, maximum pediatric dosing limits were verified using the Lexicomp drug information database, and final dosing suggestions were adjusted accordingly. This practice was consistently applied in patients who required multiple TDM assessments.

**FIGURE 2:**
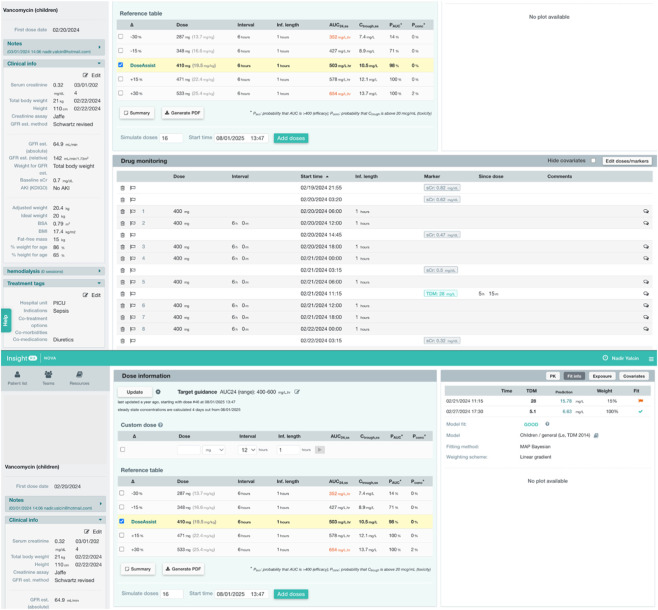
Illustrative example of vancomycin dose optimization and model fit evaluation using InsightRX.

In addition to dose optimization, InsightRX provides a model-fit evaluation for each TDM measurement. This feature assesses the agreement between observed and model-predicted drug concentrations and classifies the fit as *poor*, *intermediate*, or *good*. These classifications are based on the difference in fitted and observed concentrations and offer valuable feedback on the predictive performance of the model for each individual patient and help guide confidence in ongoing model-based dosing decisions. To facilitate better understanding of the software’s interface and functionality, a sample output from the InsightRX platform for a patient receiving vancomycin therapy is presented in Figure 2.

#### Standard of care dosing and therapeutic drug monitoring

For patients allocated to the SoC arm, InsightRX Nova® simulations were performed offline solely for research evaluation and did not influence or guide real-time dosing decisions. Clinical management in this group followed the hospital’s established therapeutic drug monitoring protocol, which relied exclusively on trough-based monitoring for both antibiotics. For vancomycin, the target trough concentration was 10–15 mg/L. For amikacin, dosing was guided by achieving trough concentrations below 5 μg/mL, in conjunction with clinical response, without the use of AUC-based calculations.

Therapeutic drug monitoring was performed according to routine institutional practice. Blood samples were typically collected at steady state—after the third or fourth dose for vancomycin and after the second or third dose for amikacin. Measured concentrations were interpreted by the attending physician in consultation with the clinical pharmacist. Dose adjustments were guided by trough concentration results and published nomograms rather than formal pharmacokinetic modeling. If trough levels were below the predefined targets, the total daily dose was proportionally increased, whereas supratherapeutic values prompted dose reduction or extension of the dosing interval. In all cases, final dosing decisions were made by the treating physician.

### Outcomes

Primary methodological outcomes were: (i) within-patient change in predictive accuracy from *a priori* to *a posteriori* prediction, and (ii) change in the model-fit category between the first and second levels. Secondary exploratory outcomes were assessed exploratorily and included treatment duration, adverse effects, mortality, and longitudinal changes in CRP, procalcitonin, and serum creatinine.

Inflammatory (CRP, procalcitonin) and renal (serum creatinine) biomarkers were collected at two predefined time points: baseline (within 24 h of antibiotic initiation) and at the time of the second TDM sample (typically day 3–5 of therapy). This schedule was applied consistently across all patients whenever serial measurements were available. To aid interpretation, both baseline and post-treatment values of CRP, procalcitonin, and serum creatinine were reported alongside their within-patient changes (Δ).

### Statistical analysis

Statistical analyses were conducted using IBM SPSS Statistics for Windows, Version 23.0 (*IBM Corp., Armonk, NY, USA*). Qualitative variables were summarized with frequency and percentage, whereas quantitative variables were summarized with median, 1^st^ quartile, 3^rd^ quartile, mean and standard deviation. Categorical variables were compared using chi-square or Fisher’s exact tests, as appropriate. For analyses involving very small or unbalanced subgroups, inferential testing was avoided and results are presented descriptively. Given the exploratory and feasibility-oriented nature of the study, multiple secondary outcomes were evaluated without formal adjustment for multiplicity. All inferential results for secondary endpoints should therefore be interpreted as exploratory and hypothesis-generating rather than confirmatory. Comparisons involving very small or unbalanced subgroups were presented descriptively without formal statistical testing. Because of the data characteristics, no adjustment for multiple comparisons was performed. Data visualization was performed using the ggplot2 (Wickham, 2016) and ggalluvial (Brunson and Read, 2023). “ggalluvial: Alluvial Plots in ‘ggplot2’.” R package version 0.12.5, http://corybrunson.github.io/ggalluvial/.’) in R packages. Differences between the administered and estimated serum levels were quantified using error metrics, including the Mean Absolute Error (MAE), $\text{Median Absolute Error }\left(\text{MdAE}\right)$ and Median Error $\left(\text{MdE}\right)$ .

For mean-based metrics:

$$\text{Mean Absolute Error }\left(\text{MAE}\right)=\frac{1}{n}\sum_{i=1}^{n}\left|{\text{predicted}}_{i}-{\text{observed}}_{i}\right|$$

For median-based metrics:

$$\text{Median Absolute Error }\left(\text{MdAE}\right)=\text{median}\left(\left|{\text{predicted}}_{i}-{\text{observed}}_{i}\right|\right)$$

$$\text{Median Error }\left(\text{MdE}\right)=\text{median}\left({\text{predicted}}_{i}-{\text{observed}}_{i}\right)$$

The symbol | | denotes absolute value; therefore, MdAE represents the median magnitude of error regardless of direction, while MdE retains the signed difference to indicate bias.

*A priori* and *a posteriori* predictive performance was assessed using paired within-patient comparisons at the same observation timepoint before and after Bayesian updating. Prediction error was quantified using MAE, MdAE, and MdE. Bootstrap confidence intervals were used for exploratory purposes and interpreted cautiously due to the limited sample size. MdAE was prespecified as the primary summary metric due to robustness to outliers.

## Results

A total of 41 pediatric patients were included in the study, with a median (IQR) age of 38.6 (81.7) months and 56.1% identified as female. The most common indication for antibiotic use was suspected or confirmed sepsis (41.46%), followed by pneumonia (29.27%) in line with ICD-11. Other less frequent indications included endocarditis (4.88%), meningitis (4.88%), and myocarditis (2.44%).

Of the 41 patients enrolled, 29 were managed with SoC and 12 underwent dose adjustment based on MIPD recommendations. The distribution of sex was similar between the two groups, with no statistically significant difference observed (p = 0.853). No statistically significant differences were found between the SoC and MIPD groups regarding treatment duration (median [IQR]: 15 [9] vs. 19 [14] days; p = 0.089), the occurrence of adverse effects (n = 1), or mortality rates (9 vs. 2 patients) (Table 2).

**TABLE 2:** Baseline demographic and clinical characteristics.

Variable	Category	SoC (N = 29)		MIPD (N = 12)		χ^2^	p-value
		n	%	n	%	​	​
Medication	Amikacin	16	55.2	2	16.7	-^a^	-^a^
	Vancomycin	13	44.8	10	83.3		
Sex	Male	13	44.8	5	41.6	0.034	0.853
	Female	16	55.2	7	58.3		
Adverse effects, n (%)	​	1	3.4	0	0	-^a^	-^a^
Mortality, n (%)	​	9	31.0	2	16.7	-^a^	-^a^
Treatment duration (days, median [IQR])	​	15 (9)		19 (14)		-	0.089
Baseline CRP (mg/L, median [IQR])	​	17.7 (18.28)		44.55 (113.5)		-	0.013
Baseline procalcitonin (ng/mL, median [IQR])	​	1.17 (2.25)		0.7 (13.76)		-	0.599
Baseline SCr (mg/dL, median [IQR])	​	0.4 (0.34)		0.46 (0.72)		-	0.663

^a^ No inferential testing was performed due to small cell counts. All reported p-values represent exploratory analyses.

Table 3 summarizes model fit results for amikacin and vancomycin. Overall, good model fits were more consistent with in the MIPD arm, particularly at the second sampling, reflecting improved predictive alignment after dose adjustment.

**TABLE 3:** Model fit evaluation and predictive performance.

Variable		Category	SoC (N = 29)		MIPD (N = 12)		χ^2^	p-value
			n	%	n	%	​	​
Model fit (1st)	Amikacin	Good	13	81.3	2	100	-^a^	-^a^
		Intermediate	2	12.5	0	0		
		Poor	1	6.3	0	0		
		Total	16	100	2	100		
	Vancomycin	Good	12	92.3	7	70	2.297	0.361
		Intermediate	0	0	1	10		
		Poor	1	7.7	2	20		
		Total	13	100	10	100		
Model fit (2nd)	Amikacin	Good	4	80	1	100	-^a^	-^a^
		Intermediate	1	20	0	0		
		Total	5	100	1	100		
	Vancomycin	good	0	0	6	100	-^a^	-^a^
		Intermediate	0	0	0	0		
		Total	0	0	6	100		

^a^ No inferential testing was performed due to small cell counts. All reported p-values represent exploratory analyses.

**FIGURE 3:**
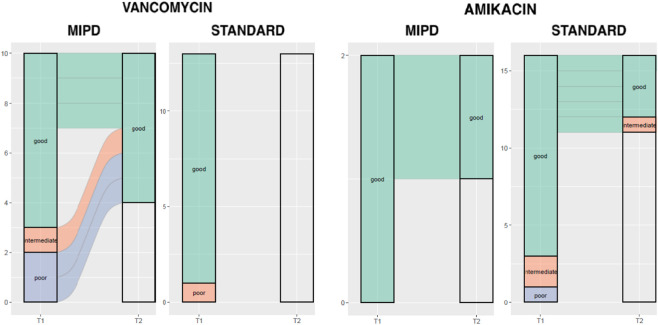
Alluvial plot of changes in pharmacokinetic model fit from T1 to T2 in MIPD and SoC Groups for Vancomycin and Amikacin MIPD: Model Informed Precision Dosing, T1: 1^st^ Serum Level-model fit category, T2: 2^nd^ serum level model fit category, x-axis: time, y-axis: counts.

Figure 3 shows the distribution of model-fit categories (poor, intermediate, good) at the first (T1) and second (T2) available TDM measurements for vancomycin and amikacin in the MIPD and standard-of-care groups. Bars reflect the number of available observations at each time point. Blank areas indicate patients without a second TDM measurement, resulting in different numbers of observations contributing to T2 across groups.

Among vancomycin users, the initial measured serum concentrations were higher in the MIPD group [median 10.0 mg/L (Q1–Q3: 8.1–18.7)] compared to the SoC group [8.7 mg/L (4.8–13.8)], although this difference was not statistically significant (p = 0.313). However, when AUC values were compared, patients in the MIPD group had higher AUC_1_ values [440 mg·h/L (398–577)] than those in the SoC group [330 mg·h/L (267–451)] (p = 0.049). This difference was expected, as the MIPD arm directly targeted AUC-based exposure while the SoC arm used trough-based monitoring. Therefore, the higher AUC observed in the MIPD group is consistent with the underlying principle of MIPD, which aims to reach therapeutic targets more rapidly and maintain them with greater consistency, rather than reflecting a methodological bias or random variation in prediction accuracy.

No significant differences were found between groups in terms of second observed or predicted serum concentrations, though the median predicted concentration was numerically higher in the MIPD group [11.56 mg/L vs. 9.36 mg/L, p = 0.166].

Inflammatory markers such as CRP and procalcitonin showed numerically greater median decreases in the MIPD group, with CRP change nonsignificant [ΔCRP: −14.75 vs. 3.25, p = 0.083].

Serum creatinine changes, as an indicator of renal function and safety, were comparable between groups (p = 0.973), indicating no additional nephrotoxicity associated with MIPD-guided dosing.

Due to the small number of amikacin users in the MIPD group (n = 2), no statistical comparisons were made, but median levels and changes in biomarkers were descriptively reported (Table 4).

**TABLE 4:** Comparison of quantitative variables in the SoC and MIPD groups for vancomycin and amikacin users.

Variable	Medication	SoC, n	SoC, median (Q1–Q3)	MIPD, n	MIPD, median (Q1–Q3)	p-value
1st Observed Concentration (mg/L)	Vancomycin	13	8.7 (4.8–13.8)	10	10 (8.1–18.7)	0.313
	Amikacin	16	0 (0–0.58)	2	1 (0–2)	-^a^
1st Predicted Concentration (mg/L)	Vancomycin	13	9.36 (5.15–13.07)	10	11.56 (9.61–15.78)	0.166
	Amikacin	16	0.71 (0.48–1.25)	2	0.48 (0.29–0.67)	-^a^
1st AUC (mg/L.hr)	Vancomycin	13	330 (267–451)	10	440 (398–577)	0.049
2nd Observed Concentration (mg/L)	Vancomycin	4	8.05 (5.5–11.4)	6	6.8 (5.1–11.4)	0.914
2nd Predicted Concentration (mg/L)	Vancomycin	2	9.62 (8.34–10.9)	6	6.57 (6.51–9.7)	-^a^
	Amikacin	6	0.88 (0.48–12.21)	1	0.77 (0.77–0.77)	-^a^
2nd AUC (mg/L.hr)	Vancomycin	3	402 (314–461)	6	291 (290–303)	-^a^0.167
ΔCRP	Vancomycin	10	3.25 (−6.6–9.81)	8	−14.75 (−81.54 to −0.915)	0.083
	Amikacin	11	2.4 (−6.6–25)	2	21.45 (−50.2–93.1)	-^a^
Δprocalcitonin	Vancomycin	9	−0.282 (−3.874 - −0.023)	6	−2.927 (−20.731 to −0.058)	0.388
	Amikacin	12	−0.197 (−2.747–0.356)	2	−0.005 (−0.168–0.158)	-^a^
ΔScr	Vancomycin	11	−0.13 (−0.33 to −0.07)	10	−0.19 (−0.58–0.03)	0.973
	Amikacin	15	−0.15 (−0.32 to −0.05)	2	0 (−0.06–0.06)	-^a^

^a^ No inferential testing was performed due to small cell counts.

AUC, area under the curve; CRP, C-reactive protein; Scr, Serum creatinine; Δ, delta, indicating the absolute change from baseline to the end of antibiotic treatment.

Mann–Whitney U test was applied where applicable.

*A priori* and *a posteriori* prediction errors represent paired within-patient comparisons at the same observation timepoint before and after Bayesian updating using the observed concentration.

**FIGURE 4:**
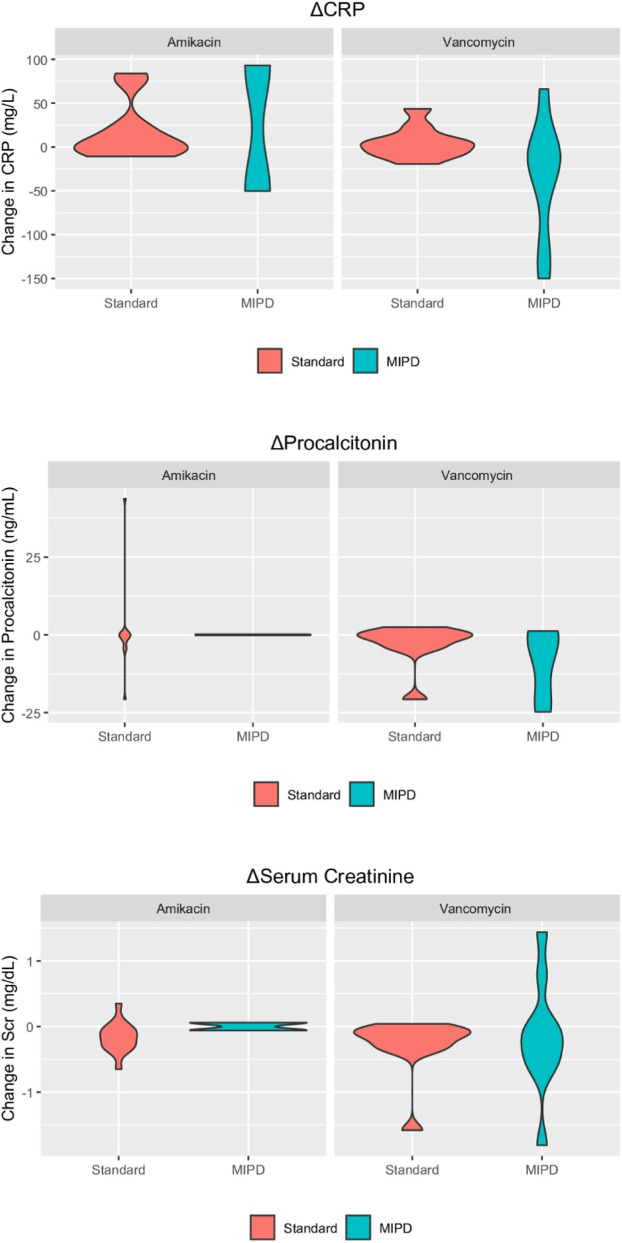
Violin plots of ΔCRP, ΔProcalcitonin and ΔSerum Creatinine for amikacin and vancomycin, Δ: delta, indicating the absolute change from baseline to the end of antibiotic treatment.

To explore potential clinical effects of MIPD-guided antibiotic therapy, changes in inflammatory and renal biomarkers were visualized using violin plots (Figure 4). Although no statistically significant differences were observed between groups, notable distributional trends emerged. Mann–Whitney U test was applied where applicable. “–” in the p-value column indicates that statistical testing was not performed due to insufficient sample size.

Among patients receiving vancomycin, median reductions in inflammatory biomarkers (CRP and procalcitonin) were numerically greater in the MIPD group compared to the SoC group, reflected by broader negative within-patient changes. These observations are exploratory in nature and should be interpreted cautiously. Given baseline imbalances and unmeasured clinical confounders, including infection severity, source control, surgical interventions, and concomitant therapies, no causal inference can be drawn regarding the effect of MIPD on inflammatory response.

Regarding serum creatinine (ΔScr), no meaningful difference was detected between the MIPD and SoC groups for either vancomycin or amikacin users, indicating that MIPD-based dosing was not associated with increased nephrotoxicity. The spread of values was visually wider in the MIPD group for vancomycin, although central tendencies remained similar.

While none of these findings reached statistical significance, these visualizations provide supportive insight into possible clinical trends that warrant further investigation in larger cohorts (Figure 4).

To assess the predictive accuracy of the pharmacokinetic models, both mean-based (MAE) and median-based (MdAE, MdE) error metrics were calculated by comparing observed versus predicted serum concentrations. Predictive performance was evaluated through within-patient paired comparisons of *a priori* (based on population priors) versus *a posteriori* (individualized via Bayesian updating) predictions at the same observation timepoints.

At the first TDM sampling point (n = 41), Bayesian updating was associated with lower prediction error across the overall cohort. The overall MAE decreased from 4.262 mg/L (*a priori*) to 2.160 mg/L (*a posteriori*), while the MdAE decreased from 1.800 mg/L to 0.730 mg/L. Corresponding error metrics stratified by treatment group and drug type are presented in Table 5.

**TABLE 5:** Evaluation of predictive performance: within-patient comparisons of a priori and a posteriori predictions.

Concentration	Group	Mean-based metrics	Median-based metrics	
		MAE	MdAE	MdE
1st TDM sample-a priori	Total (n = 41)	4.262	1.800	0.700
	MIPD (n = 12)	5.133	2.050	0.600
	SoC (n = 29)	3.902	1.600	0.700
	Amikacin users (n = 18)	2.219	0.750	0.650
	Vancomycin users (n = 23)	5.861	4.700	1.000
1st TDM sample-a posteriori	Total (n = 41)	2.160	0.730	0.250
	MIPD (n = 12)	4.511	1.760	−0.160
	SoC (n = 29)	1.187	0.680	0.300
	Amikacin users (n = 18)	0.940	0.640	0.435
	Vancomycin users (n = 23)	3.115	1.140	−0.070
2nd TDM sample-a priori	Total (n = 15)	4.530	2.000	0.550
2nd TDM sample-a posteriori (prediction using parameters updated with the 1st TDM concentration)	Total (n = 15)	2.742	0.960	0.010

In subgroup analyses, vancomycin users (n = 23) showed lower median-based error following Bayesian updating (MdAE: 4.700 mg/L *a priori* vs. 1.140 mg/L *a posteriori*). Among amikacin users (n = 18), the *a posteriori* MAE was 0.940 mg/L.

For the second TDM level (n = 15), prediction error was lower when model parameters were informed by the first TDM concentration. The *a priori* MAE and MdAE were 4.530 mg/L and 2.000 mg/L, respectively, compared with 2.742 mg/L and 0.960 mg/L for predictions at the second timepoint. Median error (MdE) values were centered near zero at both timepoints (0.250 at T1 and 0.010 at T2).

## Discussion

This study is the first to evaluate the implementation and methodological performance of MIPD in critically ill pediatric patients receiving aminoglycosides or glycopeptides in a PICU. While no statistically significant differences were observed in predicted versus measured concentrations, MIPD-guided dosing was associated with numerical differences in inflammatory markers such as CRP and procalcitonin. These observations should be interpreted cautiously and considered exploratory given the limited sample size and study design. Notably, serum creatinine levels remained comparable between groups, indicating no additional nephrotoxicity.

### Prediction accuracy

In this study the predictive accuracy of the PK model using both mean-based (MAE) and median-based (MdAE, MdE) error metrics were evaluated. Across the overall cohort, prediction error at the first TDM sampling point was reduced following Bayesian updating, with an MAE of 2.16 mg/L and an MdAE of 0.73 mg/L. These values provide a descriptive benchmark for model performance in this feasibility setting.

Prediction errors were numerically higher in the MIPD group compared with the SoC group. This difference likely reflects underlying heterogeneity, small subgroup sizes, and the exposure-based definition of analytic groups rather than a true difference in model performance. When stratified by drug type, prediction accuracy was higher for amikacin than for vancomycin, consistent with the greater pharmacokinetic variability of vancomycin in critically ill pediatric populations. Median error values were centered near zero across groups and timepoints, indicating minimal directional bias in model predictions.

Importantly, evaluation of predictive performance was based on paired within-patient comparisons of *a priori* versus *a posteriori* predictions at the same observation timepoint, rather than sequential comparisons across different sampling occasions. Within the MIPD group, paired analyses did not demonstrate a statistically significant improvement in prediction accuracy following Bayesian updating. This finding should be interpreted in the context of the study design, in which Bayesian updating was typically informed by a single routinely collected trough concentration, limiting parameter identifiability and the magnitude of observable improvement. Non-optimized sampling times driven by routine clinical practice, together with high interindividual variability in critically ill children, likely further constrained performance gains.

In contrast, prior studies evaluating MIPD performance have reported substantial gains in predictive accuracy when multiple concentrations were incorporated into Bayesian updating. For example, Heus et al. (2022) demonstrated that *a priori* predictions based only on covariates and dosing yielded RMSE values ranging from 7.0 to 22.3 mg/L across vancomycin models, whereas *a posteriori* predictions incorporating TDM data improved RMSE to 4.4–18.1 mg/L depending on the number of measurements used. Similarly, Hughes et al. (2021) reported RMSE values between 4.5 and 6.3 mg/L in PICU patients using “continual learning” models, outperforming traditional PopPK approaches.

Schatz et al. (2024) showed that integrating even a single TDM sample could significantly improve prediction precision. Their study found that model-averaging approaches outperformed individual model selection strategies, and the inclusion of a second TDM sample further enhanced accuracy and target attainment. Abdulla et al. (2021) emphasized target attainment as an indirect indicator of predictive accuracy in their review: Smits et al. (2015) reported amikacin peak and trough levels reaching target ranges in 90.5% and 60.2% of cases, respectively; Leroux et al. (2016) observed an increase in vancomycin trough attainment from 41% to 72% with MIPD; Frymoyer et al. (2017). Demonstrated a 94% AUC_24h_/MIC > 400 attainment with MIPD approach.

Ewoldt et al. (2022) evaluated MIPD’s clinical impact in critically ill adult patients receiving β-lactams and ciprofloxacin, reporting similar target attainment rates on day one (MIPD: 55.6%, SoC: 60.9%) and day three (MIPD: 59.5%, SoC: 60.4%), highlighting the need for optimized modeling strategies.

It is well established that the predictive accuracy of PK models is highly dependent on the match between the model development population and the external cohort. Factors such as patient demographics, assay methods, sampling times, and concentration ranges all influence performance. Notably, the models developed by Okada et al. (2018) and Colin et al. (2019) differ substantially in study design and patient characteristics, underscoring the variability in model applicability across settings. This underscores the need for extensive validation studies in diverse populations to identify the most generalizable models (Heus et al., 2022).

Although prediction errors were numerically higher in the MIPD arm, they remained within ranges reported in prior pediatric pharmacokinetic studies, consistent with the feasibility of implementing MIPD in pediatric critical care settings. The observed AUC differences primarily reflect the distinct PK/PD targets—AUC-guided optimization in MIPD versus trough-based adjustment in SoC—rather than intrinsic model superiority. In several patients receiving MIPD, individual model fits appeared to change following Bayesian updating, particularly for vancomycin; however, these observations are descriptive and should not be interpreted as evidence of improved predictive performance. In contrast, model fit patterns remained largely stable in the SoC group. Larger datasets with multiple TDM points are needed to confirm these observations and further refine pediatric MIPD algorithms.

### Inflammatory response trends

For transparency, both baseline and post-treatment biomarker values were presented to contextualize the observed Δ changes. Although statistical significance was not reached, reductions in inflammatory biomarkers—including CRP and procalcitonin—were more prominent in the MIPD group, especially among patients treated with vancomycin. Given baseline imbalances and unmeasured clinical confounders, these findings should be regarded as hypothesis-generating rather than indicative of causal clinical effects. CRP and procalcitonin are influenced by multiple factors beyond antibiotic exposure, including but not limited to infection severity, source control, and concomitant therapies, which were not systematically captured.

### Renal perspective with MIPD-Guided dosing

Optimizing dosing regimens for nephrotoxic antibiotics such as vancomycin and aminoglycosides requires careful consideration of renal safety. In our study, no significant differences were observed in serum creatinine changes between the MIPD-guided dosing group and the SoC group, suggesting that MIPD was not associated with increased nephrotoxicity.

Previous studies have reported more definitive outcomes. In a prospective clinical trial by Leroux et al. (2016) evaluating vancomycin dosing in neonates, the incidence of nephrotoxicity was substantially lower in the MIPD group (1.1%) compared to the SoC group (8.7%). These findings suggest that MIPD may offer a safer approach to vancomycin dosing in this vulnerable population. Similar trends have been noted in adult populations, where MIPD-guided vancomycin therapy has been associated with significantly reduced risk of acute kidney injury. These observations have motivated larger randomized controlled trials, such as the BENEFICIAL study, which aims to determine whether MIPD can reduce AKI rates in critically ill patients receiving vancomycin, as part of its tertiary outcomes (De Cock et al., 2024).

In contrast, the DOLPHIN trial by Ewoldt et al. (2022), found no significant differences in renal or hepatic toxicity between the MIPD and SoC groups. Although our findings did not demonstrate a renal benefit, existing literature suggests that MIPD may enhance renal safety, particularly with vancomycin.

### Treatment duration, mortality and adverse effects

Consistent with previous studies, no meaningful differences were observed in treatment duration or mortality between dosing strategies. These outcomes were not primary endpoints, and the study was neither powered to detect differences in hard clinical outcomes. Evidence in pediatric populations remains limited, and existing studies have primarily focused on pharmacometric endpoints rather than clinical effectiveness.

Accordingly, reported p-values for secondary outcomes are intended to provide descriptive context rather than to support definitive claims of effect. In most studies, treatment duration and mortality have not been directly evaluated as primary or secondary outcome measures in comparative analyses. For instance, in studies by Frymoyer and Bio on vancomycin dosing in pediatric patients with cystic fibrosis or in general hospitalized pediatric populations, specific comparisons of treatment duration between MIPD and SoC strategies were not reported. In Frymoyer et al. (2020), the median treatment duration among neonates and pediatric patients receiving MIPD was reported as 7 and 6 days, respectively; however, these values were not compared to a pre-MIPD control group, limiting interpretability.

The DOLPHIN trial reported a median antibiotic treatment duration of 4.1 days (IQR 1.98–6.06) in the MIPD group versus 3.5 days (IQR 2.22–5.25) in the SoC group. However, this difference was not statistically analyzed as a prespecified outcome, and thus no conclusion can be drawn regarding MIPD’s effect on treatment length (Ewoldt et al., 2022).

The same DOLPHIN trial found no significant benefit of MIPD in reducing mortality among critically ill adult patients. Rates of ICU mortality, hospital mortality, 28-day mortality, and 6-month mortality did not differ significantly between the MIPD and SoC arms.

In pediatric populations, evidence remains sparse. As highlighted by Gijsen et al. (2021), there is a notable lack of primary research directly evaluating clinical outcomes such as mortality in pediatric ICU patients receiving MIPD. While some studies have assessed target attainment rates and nephrotoxicity, they have not included mortality as a direct outcome (Hughes et al., 2021; Leroux et al., 2016).

In our study, hospital or ICU length of stay could not be assessed due to the transfer of patients to other departments prior to discharge. This limitation prevented us from capturing consistent endpoint data related to hospitalization duration. However, previous studies have attempted to explore this outcome. For example, the DOLPHIN trial designated ICU length of stay as its primary endpoint and found no statistically significant difference between groups—reporting a median of 10 days for the MIPD group and 8 days for the SoC group (IRR = 1.16; 95% CI: 0.96–1.41; p = 0.13). Although ICU stay was slightly longer in the MIPD group by 2 days, this difference was not considered clinically meaningful (Ewoldt et al., 2022).

Between-group comparisons are further limited by confounding factors, including differences in infection severity, adequacy of source control, and concomitant antimicrobial therapy, which were not controlled for in this pragmatic study design.

### Challenges in implementation and study limitations

This pilot study has several limitations that should be acknowledged. The relatively small sample size and baseline group imbalances—particularly the limited number of patients receiving amikacin in the MIPD arm—restricted statistical power and precluded more detailed subgroup analyses. These imbalances were partly attributable to the pragmatic reassignment of patients initially allocated to the MIPD arm whose prescribed doses already aligned with model recommendations and therefore did not require active intervention. Logistical constraints, including limited availability of therapeutic drug monitoring and challenges in post-treatment follow-up due to patient transfers out of the PICU, further restricted longitudinal outcome assessment. In addition, Bayesian updating was typically informed by a limited number of concentration measurements, and the original protocol did not prespecify a detailed *a priori* versus *a posteriori* prediction analysis, potentially confounding between-arm model-fit comparisons given the AUC-targeted nature of the intervention. Pharmacodynamic target attainment was evaluated using assumed MIC values due to inconsistent availability of culture results at treatment initiation, which may not reflect true pathogen susceptibility. Protein binding was not explicitly accounted for, representing a relevant limitation for vancomycin in critically ill pediatric patients with hypoalbuminemia, though less so for amikacin. Finally, renal safety assessment relied solely on serum creatinine without standardized acute kidney injury definitions, which may limit sensitivity to early renal dysfunction. Because therapeutic drug monitoring was largely trough-driven and informed by limited concentration measurements, interpretation of peak-based metrics such as Cmax/MIC should be considered exploratory. Collectively, these limitations reflect real-world implementation challenges and highlight areas for methodological refinement in future studies.

### Implications for practice and future research

As healthcare systems increasingly shift toward precision medicine, the integration of pharmacometric tools into clinical workflows offers a scalable, data-driven solution for individualized dosing.

Although the InsightRX platform used in this study accounted for patient-specific parameters such as age, weight, height, ECMO, hemodialysis, comorbidities, and concomitant medications, further multicenter popPK studies incorporating additional relevant demographic and clinical variables are warranted to enhance dosing accuracy.

An additional practical challenge encountered during implementation was clinician hesitation. As a relatively new approach, MIPD was met with some skepticism, and clinicians were initially cautious about applying model-generated recommendations. A further limitation is that clinician hesitation was only observed qualitatively and not systematically quantified; no formal questionnaire or structured assessment of participant training, profession (e.g., physicians vs. clinical pharmacists), or prior experience was performed. However, we believe that increased exposure to the system and demonstration of clinical utility in larger populations will gradually help overcome these reservations.

Furthermore, existing popPK models may vary based on population-specific characteristics, including ethnic, pharmacogenetic and regional differences. We advocate pooling and synthesis of global popPK data through meta-analyses to create more comprehensive, broadly applicable MIPD platforms. This would support the development of robust, pediatric-specific clinical decision support tools.

Future research should focus on validating our findings in larger, more diverse pediatric populations and evaluating the long-term clinical and economic outcomes of MIPD-guided dosing. Incorporating machine learning based popPK models may also enhance predictive performance and clinical applicability. Overall, our results reinforce the promise of MIPD as a transformative strategy for pediatric antibiotic therapy, provided that technical, infrastructural, and cultural barriers can be systematically addressed. This pilot study may support such research efforts, by illustrating its feasibility and providing data to support power calculations.

## Conclusion

In line with the primary objective, our findings demonstrated close agreement between model-predicted and observed serum concentrations, supporting the overall reliability of the applied pharmacokinetic models. However, improvements in prediction accuracy after Bayesian updating were not statistically significant, underscoring the need for larger datasets to confirm these results. Overall, the findings demonstrate the feasibility of implementing MIPD in pediatric intensive care, while observed trends in biomarkers and safety outcomes remain exploratory and require confirmation in larger, adequately powered studies.

## Data Availability

The data analyzed in this study is subject to the following licenses/restrictions: The datasets used and/or analyzed during the current study are available from the corresponding authors on reasonable request. Requests to access these datasets should be directed to nadir.yalcin@hacettepe.edu.tr.
